# (*E*)-2-{[(2-Amino­pyridin-3-yl)imino]­meth­yl}-4,6-di-*tert*-butyl­phenol

**DOI:** 10.1107/S1600536812032060

**Published:** 2012-07-21

**Authors:** Alexander Carreño, Sonia Ladeira, Annie Castel, Andres Vega, Ivonne Chavez

**Affiliations:** a Departamento de Ciencias Químicas, Facultad de Ciencias Exactas, Universidad Andres Bello, Programa de Doctorado en Fisico-Química Molecula, Santiago, Chile; b Université Paul Sabatier, Institut de Chimie de Toulouse (FR 2599), 118 route de Narbonne, 31062 Toulouse Cedex 9, France; c Departamento de Ciencias Químicas, Facultad de Ciencias Exactas, Universidad Andres Bello, Centro para el Desarrollo de la Nanociencia y la Nanotecnología, CEDENNA, Santiago, Chile; d Departamento de Química Inorgánica, Facultad de Química, Pontificia Universidad Católica de Chile, Santiago, Chile

## Abstract

In the title compound, C_20_H_27_N_3_O, the hy­droxy group forms an intra­molecular O—H⋯N hydrogen bond with the imino N atom. The dihedral angle between the aromatic rings is 33.09 (9)°. In the crystal, mol­ecules form centrosymmetric dimers *via* pairs of N—H⋯N hydrogen bonds involving amino­pyridine fragments.

## Related literature

For asymmetric ligands prepared from aromatic diamines and their metal complexes exhibiting catalytic activity, *e.g.* metallosalphenes, see: Kleij, Kuil *et al.* (2005 ▶); Kleij, Tooke *et al.* (2005 ▶). For the synthetic procedure, see: Benisvy *et al.* (2003 ▶, 2004 ▶). For the related structure of 2-amino-3-salicylidenamino­pyridine, see: Cimerman *et al.* (1992 ▶).
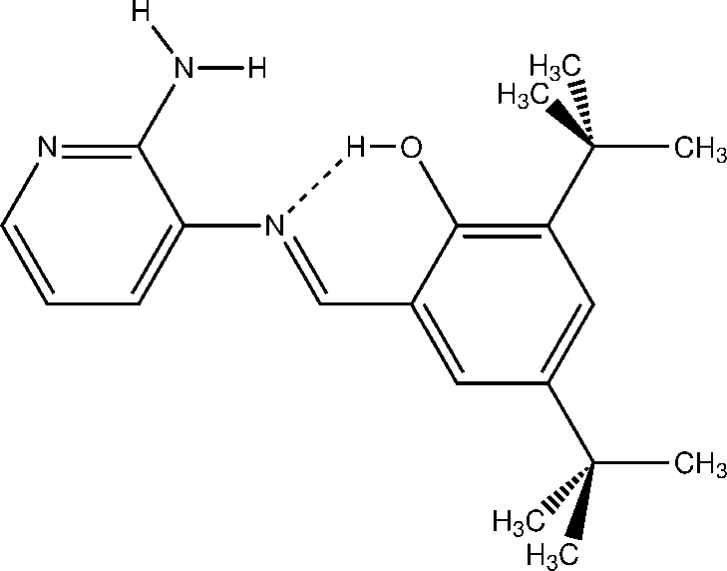


## Experimental

### 

#### Crystal data

C_20_H_27_N_3_O
*M* *_r_* = 325.45
Monoclinic, 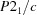

*a* = 16.8457 (12) Å
*b* = 10.6227 (8) Å
*c* = 10.4817 (6) Å
β = 101.268 (4)°
*V* = 1839.5 (2) Å^3^
*Z* = 4
Mo *K*α radiation
μ = 0.07 mm^−1^
*T* = 193 K
0.6 × 0.06 × 0.04 mm

#### Data collection

Bruker Kappa APEXII Quazar area-detector diffractometer
Absorption correction: multi-scan (*SADABS*; Bruker, 2009 ▶) *T*_min_ = 0.957, *T*_max_ = 0.997
29076 measured reflections
4532 independent reflections
2875 reflections with *I* > 2σ(*I*)
*R*_int_ = 0.063

#### Refinement

*R*[*F* ^2^ > 2σ(*F* ^2^)] = 0.054
*wR*(*F* ^2^) = 0.160
*S* = 1.03
4532 reflections
230 parameters
2 restraints
H atoms treated by a mixture of independent and constrained refinement
Δρ_max_ = 0.28 e Å^−3^
Δρ_min_ = −0.26 e Å^−3^

### 

Data collection: *APEX2* (Bruker, 2009 ▶); cell refinement: *SAINT* (Bruker, 2009 ▶); data reduction: *SAINT*; program(s) used to solve structure: *SHELXS97* (Sheldrick, 2008 ▶); program(s) used to refine structure: *SHELXL97* (Sheldrick, 2008 ▶); molecular graphics: *ORTEP-3 for Windows* (Farrugia, 1997 ▶); software used to prepare material for publication: *WinGX* (Farrugia, 1999 ▶).

## Supplementary Material

Crystal structure: contains datablock(s) global, I. DOI: 10.1107/S1600536812032060/yk2062sup1.cif

Structure factors: contains datablock(s) I. DOI: 10.1107/S1600536812032060/yk2062Isup2.hkl

Supplementary material file. DOI: 10.1107/S1600536812032060/yk2062Isup3.cml

Additional supplementary materials:  crystallographic information; 3D view; checkCIF report

## Figures and Tables

**Table 1:** Hydrogen-bond geometry (Å, °)

*D*—H⋯*A*	*D*—H	H⋯*A*	*D*⋯*A*	*D*—H⋯*A*
O1—H1*A*⋯N1	0.84	1.87	2.6214 (19)	149
N3—H203⋯N2^i^	0.89 (1)	2.16 (1)	3.045 (2)	175 (2)

Symmetry code: (i) 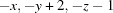.

**Fig. 1.**
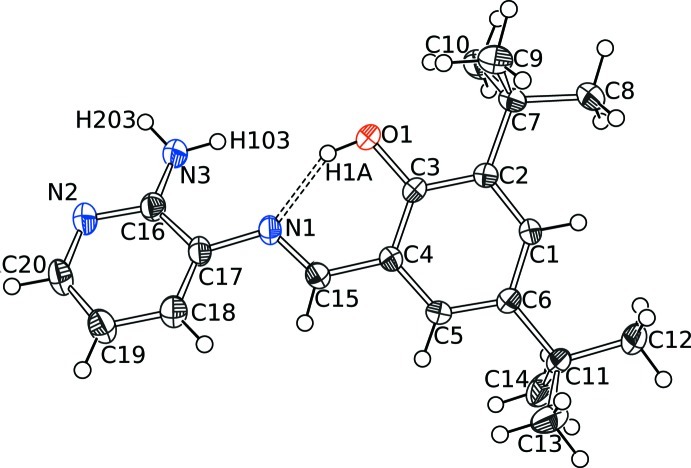
Molecular structure of the title compound showing atom labelling scheme and the intramolecular hydrogen bond. Thermal ellipsoids are drawn at the 50% probability level.

**Fig. 2.**
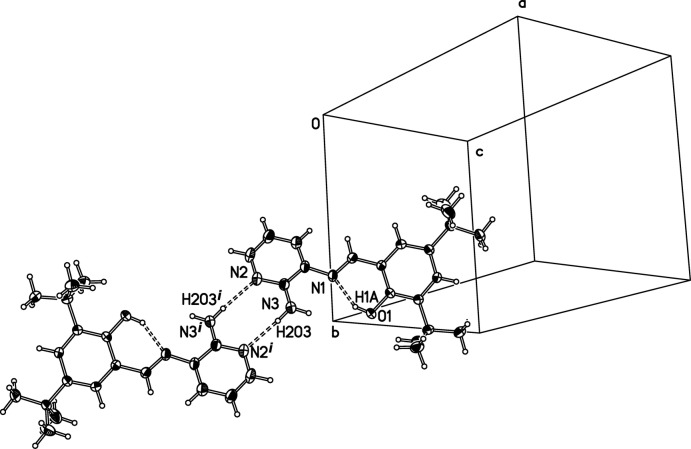
Hydrogen-bonded molecular dimers in the crystal. Symmetry code: (i) -x, 2 - y, -1 - z.

**Fig. 3.**
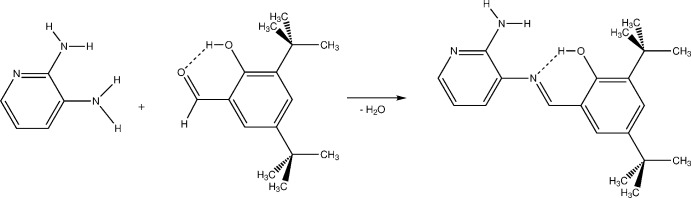
Synthetic route to the title compound.

## supplementary crystallographic information

### Comment

Aromatic diamines are used as starting materials for the templated synthesis of nonsymmetric metallosalphen complexes. These are useful in homogeneous catalysis. The non-templated synthesis pathway is also possible (Kleij, Kuil *et al.*, 2005; Kleij, Tooke *et al.*, 2005) even using diamino pyridine in combination with salicyl aldehyde derivatives. Additionaly, partially substituted products like mono Schiff bases are possible to be isolated with good yield under the same experimental conditions.

The title compound, mono Schiff base, was prepared by the non-templated direct condensation of 1,2-diaminopyridine and 3,5-di-*tert*-buthyl-2-ol-benzaldehide according to a previously described method (Benisvy *et al.*, 2003, 2004).

In the title compound, the central benzene ring is substituted at position 1 with a hydroxyl group, at positions 2 and 4 with *tert*-buthyl groups, and at position 6 with a [(2-aminopyridin-3-yl)imino]methyl. The vicinity of the hydroxyl and the [(2-aminopyridin-3-yl)imino]methyl substituents leads to an intramolecular hydrogen bond with O1···N1 distance of 2.621 (1) Å. The reported value for the similar molecule, 2-amino-3-salicylideneaminopyridine, is 2.649 (1) Å (Cimerman *et al.*, 1992).

In addition to that intramolecular interaction, the molecules form dimers in the solid state *via* hydrogen bonds between the amino pyridine fragments, as shown in Figure 2.

### Experimental

The compound was prepared by direct interaction between 1,2-diaminopyridine and 3,5-di-*tert*-butyl-2-ol-benzaldehide (Fig. 3) according to a previously described method (Benisvy *et al.*, 2003, 2004), slighty modified by using ethanol as a solvent, instead of diclorometane and 24 h as reaction time. The synthesis yield was 70%.

### Refinement

The H atoms attached to C and O were positioned geometrically and refined using a riding model, with C—H distances of 0.95 Å (CH) and 0.98 Å (CH_3_) and O—H equal to 0.84 Å. *U*_iso_(H) values were set equal to 1.5*U*_eq_ of the parent atoms for methyl and hydroxyl groups, while 1.2*U*_eq_ for the others. The amine hydrogen atoms were located in the difference Fourier map, and their coordinates were refined with N—H distances restrained to 0.88 Å.

### Crystal data

**Table:**

C_20_H_27_N_3_O	*F*(000) = 704
*M**_r_* = 325.45	*D*_x_ = 1.175 Mg m^−^^3^
Monoclinic, *P*2_1_/*c*	Mo *K*α radiation, λ = 0.71073 Å
Hall symbol: -P 2ybc	Cell parameters from 4180 reflections
*a* = 16.8457 (12) Å	θ = 2.3–24.2°
*b* = 10.6227 (8) Å	µ = 0.07 mm^−^^1^
*c* = 10.4817 (6) Å	*T* = 193 K
β = 101.268 (4)°	Needle, yellow
*V* = 1839.5 (2) Å^3^	0.6 × 0.06 × 0.04 mm
*Z* = 4	

### Data collection

**Table:**

Bruker Kappa APEXII Quazar area-detector diffractometer	4532 independent reflections
Radiation source: microfocus sealed tube	2875 reflections with *I* > 2σ(*I*)
Multilayer optics monochromator	*R*_int_ = 0.063
φ and ω scans	θ_max_ = 28.3°, θ_min_ = 3.1°
Absorption correction: multi-scan (*SADABS*; Bruker, 2009)	*h* = −22→22
*T*_min_ = 0.957, *T*_max_ = 0.997	*k* = −14→14
29076 measured reflections	*l* = −13→13

### Refinement

**Table:**

Refinement on *F*^2^	Primary atom site location: structure-invariant direct methods
Least-squares matrix: full	Secondary atom site location: difference Fourier map
*R*[*F*^2^ > 2σ(*F*^2^)] = 0.054	Hydrogen site location: inferred from neighbouring sites
*wR*(*F*^2^) = 0.160	H atoms treated by a mixture of independent and constrained refinement
*S* = 1.03	*w* = 1/[σ^2^(*F*_o_^2^) + (0.0793*P*)^2^ + 0.3155*P*] where *P* = (*F*_o_^2^ + 2*F*_c_^2^)/3
4532 reflections	(Δ/σ)_max_ = 0.001
230 parameters	Δρ_max_ = 0.28 e Å^−^^3^
2 restraints	Δρ_min_ = −0.26 e Å^−^^3^

### Special details

**Table:**

Geometry. All s.u.'s (except the s.u. in the dihedral angle between two l.s. planes) are estimated using the full covariance matrix. The cell s.u.'s are taken into account individually in the estimation of s.u.'s in distances, angles and torsion angles; correlations between s.u.'s in cell parameters are only used when they are defined by crystal symmetry. An approximate (isotropic) treatment of cell s.u.'s is used for estimating s.u.'s involving l.s. planes.
Refinement. Refinement of *F*^2^ against ALL reflections. The weighted *R*-factor *wR* and goodness of fit *S* are based on *F*^2^, conventional *R*-factors *R* are based on *F*, with *F* set to zero for negative *F*^2^. The threshold expression of *F*^2^ > σ(*F*^2^) is used only for calculating *R*-factors(gt) *etc*. and is not relevant to the choice of reflections for refinement. *R*-factors based on *F*^2^ are statistically about twice as large as those based on *F*, and *R*- factors based on ALL data will be even larger.

### Fractional atomic coordinates and isotropic or equivalent isotropic displacement parameters (Å^2^)

**Table:**

	*x*	*y*	*z*	*U*_iso_*/*U*_eq_	
C1	0.33157 (10)	0.86007 (16)	0.29538 (16)	0.0257 (4)	
H1	0.3674	0.8829	0.3736	0.031*	
C2	0.28038 (10)	0.95302 (16)	0.23052 (16)	0.0249 (4)	
C3	0.22810 (10)	0.91744 (16)	0.11465 (16)	0.0257 (4)	
C4	0.22863 (10)	0.79318 (16)	0.06788 (16)	0.0245 (4)	
C5	0.28054 (10)	0.70461 (16)	0.13891 (17)	0.0266 (4)	
H5	0.2794	0.6204	0.108	0.032*	
C6	0.33349 (10)	0.73537 (16)	0.25259 (16)	0.0253 (4)	
C7	0.28219 (12)	1.08949 (17)	0.28088 (17)	0.0320 (4)	
C8	0.34609 (13)	1.10793 (19)	0.40455 (18)	0.0390 (5)	
H8A	0.3332	1.0544	0.4739	0.058*	
H8B	0.3467	1.1963	0.4315	0.058*	
H8C	0.3994	1.0849	0.3876	0.058*	
C9	0.19959 (13)	1.1247 (2)	0.3122 (2)	0.0461 (5)	
H9A	0.157	1.1094	0.2357	0.069*	
H9B	0.1997	1.2139	0.336	0.069*	
H9C	0.1894	1.0733	0.385	0.069*	
C10	0.30327 (16)	1.17916 (19)	0.1770 (2)	0.0478 (6)	
H10A	0.355	1.154	0.1554	0.072*	
H10B	0.3075	1.2654	0.2109	0.072*	
H10C	0.2607	1.1752	0.0987	0.072*	
C11	0.39232 (11)	0.63577 (17)	0.32438 (16)	0.0304 (4)	
C12	0.43933 (14)	0.6829 (2)	0.4540 (2)	0.0479 (6)	
H12A	0.4739	0.6152	0.4976	0.072*	
H12B	0.4014	0.7094	0.5088	0.072*	
H12C	0.4731	0.7546	0.4394	0.072*	
C13	0.34424 (14)	0.5187 (2)	0.3506 (2)	0.0473 (6)	
H13A	0.3133	0.4864	0.2681	0.071*	
H13B	0.307	0.5414	0.408	0.071*	
H13C	0.3818	0.4537	0.3923	0.071*	
C14	0.45191 (13)	0.5987 (2)	0.2383 (2)	0.0484 (6)	
H14A	0.422	0.5655	0.1555	0.073*	
H14B	0.4889	0.534	0.2823	0.073*	
H14C	0.4831	0.6728	0.2218	0.073*	
C15	0.17797 (10)	0.75478 (17)	−0.05444 (16)	0.0275 (4)	
H15	0.1788	0.6688	−0.079	0.033*	
C16	0.05978 (11)	0.86736 (18)	−0.35148 (17)	0.0299 (4)	
C17	0.08167 (10)	0.78338 (17)	−0.24490 (16)	0.0281 (4)	
C18	0.04954 (11)	0.66344 (19)	−0.25690 (18)	0.0335 (4)	
H18	0.0632	0.605	−0.1874	0.04*	
C19	−0.00259 (12)	0.6285 (2)	−0.37037 (18)	0.0383 (5)	
H19	−0.0231	0.5451	−0.3819	0.046*	
C20	−0.02377 (12)	0.7174 (2)	−0.46567 (18)	0.0397 (5)	
H20	−0.0615	0.6942	−0.5417	0.048*	
N1	0.13236 (9)	0.83038 (15)	−0.13087 (14)	0.0289 (4)	
N2	0.00551 (10)	0.83493 (16)	−0.45811 (14)	0.0355 (4)	
N3	0.09503 (10)	0.98206 (16)	−0.35261 (16)	0.0369 (4)	
H103	0.1261 (11)	1.010 (2)	−0.2805 (14)	0.044*	
H203	0.0683 (12)	1.0386 (16)	−0.4065 (17)	0.044*	
O1	0.17780 (8)	1.00391 (12)	0.04615 (12)	0.0353 (3)	
H1A	0.1527	0.9712	−0.023	0.053*	

### Atomic displacement parameters (Å^2^)

**Table:**

	*U*^11^	*U*^22^	*U*^33^	*U*^12^	*U*^13^	*U*^23^
C1	0.0288 (9)	0.0250 (9)	0.0211 (8)	−0.0005 (7)	−0.0006 (7)	−0.0018 (7)
C2	0.0281 (9)	0.0224 (9)	0.0234 (8)	0.0010 (7)	0.0029 (7)	−0.0004 (7)
C3	0.0268 (9)	0.0238 (9)	0.0245 (8)	0.0038 (7)	0.0001 (7)	0.0028 (7)
C4	0.0253 (9)	0.0225 (9)	0.0241 (8)	−0.0017 (7)	0.0006 (7)	−0.0012 (6)
C5	0.0296 (9)	0.0212 (9)	0.0275 (9)	0.0013 (7)	0.0025 (7)	−0.0020 (7)
C6	0.0266 (9)	0.0246 (9)	0.0236 (8)	0.0027 (7)	0.0025 (7)	0.0023 (7)
C7	0.0436 (11)	0.0215 (9)	0.0292 (9)	0.0018 (8)	0.0025 (8)	−0.0033 (7)
C8	0.0523 (12)	0.0277 (10)	0.0331 (10)	−0.0017 (9)	−0.0009 (9)	−0.0065 (8)
C9	0.0538 (13)	0.0368 (12)	0.0456 (12)	0.0138 (10)	0.0043 (10)	−0.0092 (9)
C10	0.0753 (16)	0.0253 (10)	0.0383 (11)	−0.0091 (11)	0.0004 (11)	0.0029 (9)
C11	0.0374 (10)	0.0268 (9)	0.0243 (9)	0.0097 (8)	−0.0007 (7)	0.0006 (7)
C12	0.0552 (13)	0.0433 (13)	0.0358 (11)	0.0196 (11)	−0.0141 (10)	−0.0033 (9)
C13	0.0563 (14)	0.0303 (11)	0.0533 (13)	0.0066 (10)	0.0060 (11)	0.0123 (10)
C14	0.0483 (13)	0.0567 (14)	0.0394 (12)	0.0260 (11)	0.0067 (10)	0.0056 (10)
C15	0.0271 (9)	0.0266 (9)	0.0272 (9)	−0.0002 (7)	0.0016 (7)	−0.0039 (7)
C16	0.0288 (9)	0.0342 (10)	0.0254 (9)	0.0033 (8)	0.0024 (7)	−0.0034 (7)
C17	0.0246 (9)	0.0348 (10)	0.0229 (9)	0.0011 (8)	0.0002 (7)	−0.0038 (7)
C18	0.0319 (10)	0.0374 (11)	0.0296 (9)	−0.0020 (8)	0.0020 (8)	0.0001 (8)
C19	0.0386 (11)	0.0407 (12)	0.0334 (10)	−0.0104 (9)	0.0014 (8)	−0.0041 (9)
C20	0.0397 (11)	0.0483 (13)	0.0270 (10)	−0.0079 (10)	−0.0037 (8)	−0.0072 (9)
N1	0.0273 (8)	0.0332 (9)	0.0234 (7)	0.0003 (6)	−0.0016 (6)	−0.0025 (6)
N2	0.0363 (9)	0.0400 (10)	0.0263 (8)	−0.0014 (7)	−0.0035 (7)	−0.0021 (7)
N3	0.0436 (10)	0.0318 (9)	0.0298 (9)	0.0002 (8)	−0.0063 (7)	0.0015 (7)
O1	0.0397 (8)	0.0274 (7)	0.0321 (7)	0.0098 (6)	−0.0093 (6)	−0.0004 (5)

### Geometric parameters (Å, º)

**Table:**

C1—C2	1.397 (2)	C11—C13	1.538 (3)
C1—C6	1.401 (2)	C12—H12A	0.98
C1—H1	0.95	C12—H12B	0.98
C2—C3	1.406 (2)	C12—H12C	0.98
C2—C7	1.541 (2)	C13—H13A	0.98
C3—O1	1.356 (2)	C13—H13B	0.98
C3—C4	1.409 (2)	C13—H13C	0.98
C4—C5	1.396 (2)	C14—H14A	0.98
C4—C15	1.453 (2)	C14—H14B	0.98
C5—C6	1.381 (2)	C14—H14C	0.98
C5—H5	0.95	C15—N1	1.279 (2)
C6—C11	1.541 (2)	C15—H15	0.95
C7—C8	1.527 (2)	C16—N2	1.343 (2)
C7—C9	1.537 (3)	C16—N3	1.357 (3)
C7—C10	1.539 (3)	C16—C17	1.421 (2)
C8—H8A	0.98	C17—C18	1.380 (3)
C8—H8B	0.98	C17—N1	1.417 (2)
C8—H8C	0.98	C18—C19	1.384 (3)
C9—H9A	0.98	C18—H18	0.95
C9—H9B	0.98	C19—C20	1.370 (3)
C9—H9C	0.98	C19—H19	0.95
C10—H10A	0.98	C20—N2	1.339 (3)
C10—H10B	0.98	C20—H20	0.95
C10—H10C	0.98	N3—H103	0.882 (9)
C11—C12	1.518 (3)	N3—H203	0.885 (9)
C11—C14	1.527 (3)	O1—H1A	0.84
			
C2—C1—C6	124.28 (15)	C12—C11—C6	112.56 (15)
C2—C1—H1	117.9	C14—C11—C6	108.83 (15)
C6—C1—H1	117.9	C13—C11—C6	109.36 (15)
C1—C2—C3	116.96 (15)	C11—C12—H12A	109.5
C1—C2—C7	121.96 (15)	C11—C12—H12B	109.5
C3—C2—C7	121.07 (15)	H12A—C12—H12B	109.5
O1—C3—C2	119.77 (15)	C11—C12—H12C	109.5
O1—C3—C4	119.75 (15)	H12A—C12—H12C	109.5
C2—C3—C4	120.47 (15)	H12B—C12—H12C	109.5
C5—C4—C3	119.45 (15)	C11—C13—H13A	109.5
C5—C4—C15	118.68 (15)	C11—C13—H13B	109.5
C3—C4—C15	121.84 (15)	H13A—C13—H13B	109.5
C6—C5—C4	122.16 (16)	C11—C13—H13C	109.5
C6—C5—H5	118.9	H13A—C13—H13C	109.5
C4—C5—H5	118.9	H13B—C13—H13C	109.5
C5—C6—C1	116.65 (15)	C11—C14—H14A	109.5
C5—C6—C11	120.27 (15)	C11—C14—H14B	109.5
C1—C6—C11	123.06 (15)	H14A—C14—H14B	109.5
C8—C7—C9	107.74 (16)	C11—C14—H14C	109.5
C8—C7—C10	107.40 (17)	H14A—C14—H14C	109.5
C9—C7—C10	110.13 (17)	H14B—C14—H14C	109.5
C8—C7—C2	112.01 (15)	N1—C15—C4	123.62 (16)
C9—C7—C2	110.13 (16)	N1—C15—H15	118.2
C10—C7—C2	109.38 (15)	C4—C15—H15	118.2
C7—C8—H8A	109.5	N2—C16—N3	116.81 (16)
C7—C8—H8B	109.5	N2—C16—C17	121.54 (17)
H8A—C8—H8B	109.5	N3—C16—C17	121.60 (16)
C7—C8—H8C	109.5	C18—C17—N1	124.26 (16)
H8A—C8—H8C	109.5	C18—C17—C16	118.10 (16)
H8B—C8—H8C	109.5	N1—C17—C16	117.58 (16)
C7—C9—H9A	109.5	C17—C18—C19	119.84 (18)
C7—C9—H9B	109.5	C17—C18—H18	120.1
H9A—C9—H9B	109.5	C19—C18—H18	120.1
C7—C9—H9C	109.5	C20—C19—C18	118.20 (19)
H9A—C9—H9C	109.5	C20—C19—H19	120.9
H9B—C9—H9C	109.5	C18—C19—H19	120.9
C7—C10—H10A	109.5	N2—C20—C19	123.99 (17)
C7—C10—H10B	109.5	N2—C20—H20	118
H10A—C10—H10B	109.5	C19—C20—H20	118
C7—C10—H10C	109.5	C15—N1—C17	119.76 (16)
H10A—C10—H10C	109.5	C20—N2—C16	118.12 (17)
H10B—C10—H10C	109.5	C16—N3—H103	118.8 (14)
C12—C11—C14	109.03 (17)	C16—N3—H203	116.5 (14)
C12—C11—C13	107.93 (17)	H103—N3—H203	118 (2)
C14—C11—C13	109.08 (17)	C3—O1—H1A	109.5

### Hydrogen-bond geometry (Å, º)

**Table:**

*D*—H···*A*	*D*—H	H···*A*	*D*···*A*	*D*—H···*A*
O1—H1*A*···N1	0.84	1.87	2.6214 (19)	149
N3—H203···N2^i^	0.89 (1)	2.16 (1)	3.045 (2)	175 (2)

Symmetry code: (i) −*x*, −*y*+2, −*z*−1.
